# Spatial-Temporal Responses of Ecosystem Services to Land Use Transformation Driven by Rapid Urbanization: A Case Study of Hubei Province, China

**DOI:** 10.3390/ijerph19010178

**Published:** 2021-12-24

**Authors:** Xufeng Cui, Cuicui Liu, Ling Shan, Jiaqi Lin, Jing Zhang, Yuehua Jiang, Guanghong Zhang

**Affiliations:** School of Business Administration, Zhongnan University of Economics and Law, Wuhan 430073, China; cxf@zuel.edu.cn (X.C.); lcc@stu.zuel.edu.cn (C.L.); shanling@stu.zuel.edu.cn (L.S.); linjiaqi@stu.zuel.edu.cn (J.L.); zjing@stu.zuel.edu.cn (J.Z.)

**Keywords:** urbanization, land use transformation, ecosystem services value, spatial autocorrelation, hot spot analysis, Hubei province, gravity model

## Abstract

Exploring the changes of ecosystem services value caused by land use transformation driven by urbanization is crucial for ensuring the safety of the regional ecological environment and for enhancing the value of ecosystem services. Based on the land use remote sensing data during the rapid urbanization development period of Hubei Province from 1995 to 2015, this study analyzed the characteristics of land use/land cover change and land use transformation. The spatial–temporal response characteristics and evolution of ecosystem services value (ESV) to land use transformation driven by urbanization were measured by equivalent factor method, spatial autocorrelation analysis, hot spot analysis and gravity model. We found that: (1) Driven by urbanization, the most significant feature of land use transformation in Hubei Province was the expansion of the built-up land and the significant reduction of cropland and forest, among which 90% of the new built-up land was converted from cropland and forest. (2) This land use transformation became the main source of ESV losses. Especially, the sharp increase of the built-up land from 2010 to 2015, occupying cropland and forest, resulted in ESV losses of nearly USD 320 million. The service capacity of climate regulation, soil conservation, gas regulation and food production undertaken by cropland and forest decreased. (3) The ecosystem services value in the study area showed spatial distribution characteristics of high in the west and low in the middle and east regions. The center of gravity of ESV shifted from northwest to southeast. Due to the sharp increase of the built-up land from 2010 to 2015, the center of gravity shift rebounded. This study can help policymakers better understand the trade−offs between land use transformation and ecosystem services driven by urbanization.

## 1. Introduction

With the continuous development of the world economy, the process of global urbanization is accelerating. According to the Revised World Urbanization Prospects 2018, 55% of the world’s population lives in cities, and this proportion will reach 68% by 2050 [1]. From 2015 to 2030, Asia and Africa will account for 90% of the global urban population growth (equivalent to 2.5 billion people), of which China, India and Nigeria will contribute 37% [2]. Unlike countries such as North America and Brazil, which have reached at least 80% urbanization level, most countries in Africa and Asia are experiencing rapid urbanization [3]. Rapid urbanization has brought qualitative changes to the economic, administrative, cultural and social aspects of cities, but it has also increased the pressure on the ecosystem and its components, such as natural resources environment and land use change [4]. For example, Brazil entered a period of rapid urbanization development in the 1950s, and the urbanization level increased from 36% in 1950 to 65% in 1980. However, with rapid urban development, economic, social and environmental problems also appeared, such as underemployment, urban disorder development and environmental pollution [5,6]. China has experienced the largest and fastest urbanization process. According to the data of China Bureau of statistics, the urbanization level has risen from 17.90% in 1978 to 63.89% in 2020. In this process, although the acceleration of urbanization promotes social and economic development [7], it also leads to irreversible changes in land use [7,8], mainly because the significant increase in urban land leads to a large amount of occupation of natural land such as cropland, wetland and grassland [9]. In recent decades, the pattern of land use/land cover in Mainland China has undergone significant changes [10], and urbanization is one of the most important reasons for this change.

Land use changes reflect the most direct interaction between human activities and ecosystems [11,12], and are therefore regarded as the main driving factor of ecological processes and ecosystem services [13]. Abundant research has confirmed a certain connection between land use/land cover change and ecosystem services [14,15]. For example, the replacement of forest and pasture land by cultivated land affects the soil regulation function of the ecosystem [16]. Land use change affects the food production function of the ecosystem [17,18,19]. The expansion of industrial and residential land has affected the water supply function of the ecosystem [20,21,22], resulting in water resource shortages [23]. Land use/land cover change can change the surface−atmosphere interaction in energy exchanged and have an additional effect on temperature, thus affecting the climate regulation function of ecosystem [24,25]. It also affects regional biodiversity at multiple scales and lead to changes in landscape structure [26,27,28]. These studies have proved that land use change causes changes in the structure and function of the ecosystem, thus changing the provision of ecosystem services.

Ecosystem services refer to the benefits obtained from ecosystems that directly or indirectly support human survival and development, including provisioning, regulation, supporting and cultural services, which are related to human life, health and well−being [29,30,31]. Since ecosystem services are crucial to maintaining the quality of human life, many scholars have devoted themselves to estimating and evaluating the value of ecosystem services. There are many methods to evaluate the value of ecosystem services, including monetary model, investment model [32,33], shadow engineering [34], matrix method [35], etc. Among them, the currency model evaluates the value of ecosystem services in monetary units from the perspective of economic benefits [36]. In 1997, based on the equilibrium value theory and effect to value theory, Costanza formulated the equivalent factor table of global ecosystem service value, determined the first global coefficient of ESV, and measured the ESV change caused by land use change in the perspective of monetary units [29]. On the basis of Costanza’s results and a questionnaire of 500 Chinese ecologists, Chinese scholar Xie Gaodi determined the ESV equivalent factors suitable for China’s terrestrial ecosystem, and based on China’s national conditions through the biomass parameter tree equivalent factor table for multiple revisions to formulate “Chinese terrestrial ecosystem service value equivalent factor table” [37,38,39]. At present, measuring the impact of land use/cover change on the value of ecosystem services has become an important field of sustainable development science [40]. In recent years, many scholars have carried out extensive research on this in various regions, such as the world [41,42], Africa [43] OECD countries [44], Latin America and the Caribbean [45], China [46], Nigeria [14], Nepal [47], India [48], Lake Balaton in Central Europe [49], China’s Hengduan Mountains [50], Qinghai−Tibet Plateau [51], the Middle Yangtze River City Group [52], Ethiopian Plateau [53], Coastal areas around the Bohai Sea [54]. These are enough to show that all regions in the world pay more and more attention to ecosystems. It is widely used to measure the health status of ecosystem through the value of ecosystem services.

In recent years, there has been considerable research on land use change and ecosystem services, which can be divided into two categories: The first type is based on different models, such as CLUE−S model [55], CA−Markov model [56], etc., based on land use conditions in different scenarios to predict the impact of future land use changes on the value of ecosystem services. These scenarios generally include three scenarios: natural growth scenario, economic development scenario, and ecological protection scenario. The other is to use land use and cover data to explore the temporal and spatial changes of the ecosystem and discover the factors that lead to this change. These factors are mostly natural and man−made factors [33,57,58,59] and policy factors [60]. The results of many scholars on the relationship between land use change and ecosystem services have enriched the research in the ES field. However, there are few studies on the impact of rapid urbanization on land use and ecosystem services. “Urbanization−Land Use Change−Ecosystem Services Value” is a coherent response process. In the region with rapid urbanization, this research is necessary to enhance the public’s understanding of ecosystem service value and provide policy support for the government. The urbanization rate of Hubei province was 31.20% in 1995 and 56.85% in 2015. With an average annual growth rate of 1.28 percentage points in the past 20 years, the urbanization rate has increased by 25.65 percentage points, which is much faster than the urbanization development rate of developed countries and regions in Europe and the United States at the same stage. The rapid development of urbanization leads to urban expansion, non−agriculturalization of cropland, increase of transportation facilities, and reduction of lakes, all of which affect land use patterns and lead to land use/land cover change. How to assess the effects of these land use changes? The response to the value of its ecosystem services is an important evaluation path. Therefore, this research attempts: (1) To investigate the changes of LULC from 1995 to 2015 as the research basis and observe the characteristics of land use transformation in Hubei. (2) To measure the changes of ESV and ecosystem services in Hubei province from 1995 to 2015. (3) To explore the spatial response mode of ecosystem services value from the perspective of county. (4) In general, to reveal the response patterns of ecosystem services value to land use transformation driven by urbanization.

## 2. Materials and Methods

### 2.1. Study Area

**Figure 1:**
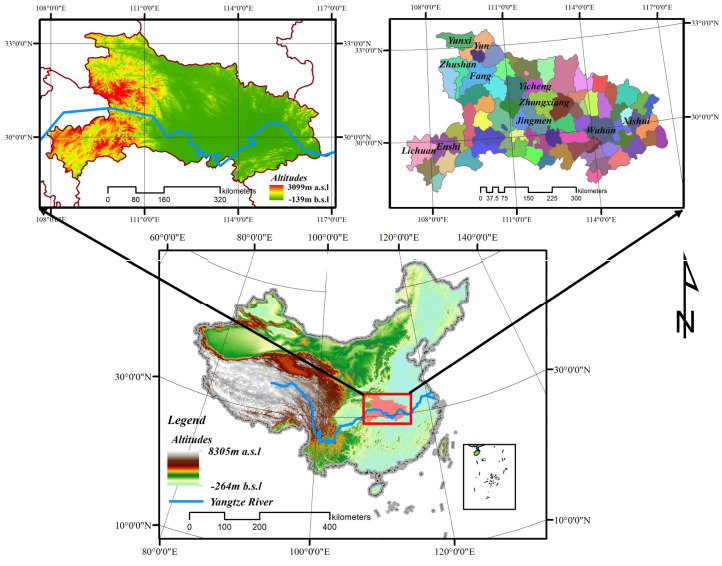
Location of Hubei, China. Note: Figure in top right shows the administrative area of Hubei Province. It consists of 77 administrative units, including 12 prefecture level cities, 1 Autonomous Prefecture, 26 county−level cities, 35 counties, 1 Autonomous County and 1 forest area.

Hubei province is located in central China, in the central of the Yangtze River basin, north of Dongting Lake (118°21′42″–116°07′50″ E, 29°01′53″–33°6′47″ N) and is rich in water resources. Administratively, the province has 12 prefecture−level cities, 1 autonomous prefecture, 26 county−level cities, 35 counties and 1 forest area, covering a total area of 185,900 km^2^ (Figure 1). By 2020, the permanent population of Hubei had reached 59.27 million, an increase of 755,000 over 2015. Hubei province has developed industry, and equipment manufacturing industry is its important pillar industry. In recent years, it has paid attention to green environmental protection and vigorously developed tourism, low−carbon industry and the new energy industry. From the perspective of industrial structure, the proportion of tertiary industry has risen in recent years. Hubei has a variety of landforms, consisting of mountains, hills, plains and lakes. Because it is located in the transition zone of the second step to the third step of China’s terrain, the terrain is high on three sides, and the middle is low and flat. Most of the region has a subtropical monsoon humid climate with an average annual temperature of 15°~17°and an average annual rainfall of 800~1600 mm. The superior natural geographical environment breeds and preserves rich and diverse biological communities, animal and plant resources, including many rare and endangered species and national key protected species. Among them, there are 1300 species of woody plants, many tree species, and precious and rare relict plants are preserved. Located in the Yangtze River basin, Hubei province has 206 species of fish [61]. The central and southern Jianghan Plain along the river is the economic center of Hubei province. The western and eastern parts of Hubei are mostly high mountains and large rivers. They are located around Hubei province with underdeveloped transportation and relatively backward economic development. During the study period, the urbanization rates in Hubei province increased from 31.20% to 56.85%. With the rapid development of urbanization in Hubei province, a series of ecological problems, such as the shortage of urban green space, environmental pollution, land shortage and biodiversity reduction, have emerged along with the transformation of land use [62].

### 2.2. Data Sources

This study used the LULC datasets from the 1995−2015 provided by the Data Center for Resources and Environmental Sciences of the Chinese Academy of Sciences (http://www.resdc.cn/, accessed on 15 December 2021). Using Landsat TM/ETM remote sensing images as the data source, the raster data onto national land use types of 1995, 2000, 2005, 2010 and 2015 with 1 km spatial resolution were generated by human–machine interactive interpretation. This paper adopted the LULC classification system and Xie Gaodi’s [63] method of classifying terrestrial ecosystem in China, combines the land use characteristics of Hubei province, and divides the land use types into seven categories: cropland, forest land, grassland, water, wetland, bare land and built-up land, then adjusts and consolidates the secondary classification. The crop production, sown area and crop price data required for the study were obtained from the Hubei Statistical Yearbook 2016, the National Agricultural Product Price Survey Yearbook 2016, and the National Compilation of Information on Costs and Returns of Agricultural Products 2016.

### 2.3. Methods

#### 2.3.1. Calculation of ESV

The ecosystem services classification method proposed by Costanza et al. cannot be directly applied to the calculation of ecosystem service value of China. This paper refers to the equivalent factor method and revised equivalent factor table based on Chinese ecosystem services [39], and modifies it by using grain yield correction and biomass correction methods in combination with the actual production capacity in Hubei province. With an average yield of 1 hm^2^, 1/7 of the annual economic value of natural grain yields to cropland is a standard ecosystem service value equivalent factor [37].

The specific correction process is as follows: Select early rice, medium rice, late rice, japonica rice, wheat and corn as the main food crops. By consulting Hubei Statistical Yearbook in 2016 and the National Compilation of Information on Costs and Returns of Agricultural Products, the grain crops to yield, sown area and grain price and output value of Hubei province in 2015 are obtained. According to Formula (1), the equivalent value of a single ecosystem in Hubei province is 2516.20 CNY/hm^2^. The ecosystem services value coefficients are shown below (Table 1).

(1) $$E_{n}=\frac{1}{7}\overset{n}{\underset{i=1}{\sum }}\frac{q_{i}p_{i}}{M}$$

where $E_{n}$ is the economic value (USD/hm^2^) of 1 hm^2^ ecosystem service value equivalent factor, *i* is the type of food crops in the study area; *n* is the total number of major food crops in the study area; $p_{i}$ is the average price of the crop *i* in the study area. $\;q_{i}$ is the average yield per unit area of type *i* food crop in the study area, and M is the total sown area of all food crops in the study area.

On this basis, according to the area of each land use type, the ecosystem services value of Hubei province can be calculated as follows [38,56]:

(2) $$ESV=\sum^{\;}A_{i}\times VC_{i}$$

(3) $$ESV_{f}=\sum^{\;}\left(A_{i}\times VC_{if}\right)$$

where ESV is the service value of the ecosystem in the study area, $ESV_{f}$ represents the value of the service function for species *f*, and $A_{i}$ is the area of type *i* land use type. $VC_{i}$ is the ecosystem services value coefficient of type *i* land use type, and $VC_{if}$ is the value of the *f*th ecological service function of the *i*th land use type.

#### 2.3.2. Land Use Transfer Matrix

Land use transfer matrix is the application of Markov model in land use change. It can show the transfer direction and quantitative change of different land use types and reveal the evolution process of land use pattern. It comes from the quantitative description of system state and state transfer in system analysis. In Formula (4), $S_{\left(t\right)}$ is the system state at time *t*, $S_{\left(t+1\right)}$ is the system state at time *t* + 1, $P_{ij}$ is the transition probability matrix in the state, and the calculation formula is as Formula (5) [64,65]:

(4) $$S_{\left(t,t+1\right)}=P_{ij}\times S_{\left(t\right)}$$

(5) $$P_{ij}=\left[\begin{array}{cc} \begin{array}{ccc} P_{11} & P_{12} & \cdots \\ P_{21} & P_{22} & \ldots \\ \vdots & \vdots & \vdots \end{array} & \begin{array}{c} P_{1n}\\ P_{2n}\\ \vdots \end{array}\\ \begin{array}{ccc} P_{n1} & P_{n2} & \ldots \end{array} & P_{nn} \end{array}\right]\;\left(0\leq P_{ij}\leq 1\right)$$

where *P* is Markov probability matrix, $P_{ij}$ represents the probability of transferring from the current state *i* to another state *j* in the next time period. The low conversion probability is close to 0 and the high conversion probability is close to 1.

In this research, after reclassifying the land use type grid data with ArcGIS10.2 software, import it into ENVI software, create ENVI standard classification format, edit the header file, and obtain the land use transfer matrix every 5 years through the Change Detection Statistics tool.

#### 2.3.3. Spatial Autocorrelation Analysis

Spatial autocorrelation analysis aims to reveal the correlation and difference between the spatial distribution of a certain attribute and its neighboring regions. If the spatial correlation is positive, it indicates that the spatial distribution of the attribute has agglomeration effect. If the spatial correlation is negative, it indicates that the spatial distribution of the attribute value is significantly different. Spatial autocorrelation is divided into global spatial autocorrelation and local spatial autocorrelation. This study uses the global autocorrelation model to determine the spatial distribution pattern of ecosystem service value. The Global Moran’s *I* index is usually used for this calculation, and the formula is as follows [66,67]:

(6) $$I=\frac{\sum_{i=1}^{n}\sum_{j=1}^{n}W_{\mathrm{ij}}\left(x_{i}-\bar{x}\right)\left(x_{j}-\bar{x}\right)}{S^{2}\sum_{i=1}^{n}\sum_{j=1}^{n}W_{\mathrm{ij}}},\;I\in \left[-1,\;1\right]$$

where *I* is Moran index. $x_{i}$ and $x_{j}$ are the observed values of the *i*th evaluation unit and the *j*th ESV evaluation unit, respectively. $W_{ij}$ is the spatial weight matrix between unit *i* and unit *j*.$\;\bar{x}$ is the mean of the observed values. n is the sample size, that is, the total number of ESV evaluation units at a certain scale in the study area. The Global Moran’s *I* index is between −1 to 1. When the value is closer to −1, it indicates that the greater the difference between evaluation units, the stronger the negative correlation, and the more discrete the spatial distribution of ESV. When the Global Moran’s *I* index value is closer to 1, it indicates that the attribute difference between evaluation units is smaller, the positive correlation is stronger, and the spatial distribution of ESV is more concentrated. When the Global Moran’s *I* index value is close to 0, it indicates that there is no correlation between evaluation units, and the spatial distribution of ESV is random.

Hot spot analysis is a common tool to identify the spatial distribution of cold spots and hot spots. Using the Getis−Ord G* index to analyze the local correlation between cold spots and hot spots can further study the local performance of the change of ecosystem service value in space. Hot spot analysis can determine whether there are high−value clusters (hot spots) and low−value clusters (cold spots) in the ecosystem service value of Hubei Province, as well as the spatial clustering positions of high−value and low−value regions [68]. By calculating the score Z (standard deviation) and probability P between each patch, the spatial location where the high−value elements and low−value elements gather [33].

(7) $$G_{i}^{*}=\frac{\sum_{j=1}^{n}\omega_{ij}x_{j}-\bar{x}\sum_{j=1}^{n}\omega_{ij}}{S\times \sqrt{\frac{{\left[n\sum_{j=1}^{n}\omega_{ij}^{2}-\sum_{j=1}^{n}\omega_{ij}\right]}^{2}}{n-1}}}$$

(8) $$\bar{x}=\frac{\sum_{j=1}^{n}x_{j}}{n}$$

(9) $$S=\sqrt{\frac{\sum_{j=1}^{n}x_{j}^{2}}{n-1}-{\bar{x}}^{2}}$$

$x_{j}$ is plaque attribute value, and $\omega_{ij}$ is the spatial weight matrix between plaque *i* and plaque *j*. *n* is the total number of plaques; $G_{i}^{*}$ is the Z score, and the Z score is a measure of statistical significance. The higher or the lower the value of $G_{i}^{*}$, the tighter the accumulation of hot spots or cold spots. When the ESV value is much larger than the adjacent area, a statistically significant hot spot is formed, which is called the hot spot area, that is, the area with high ESV change value. When the ESV value is much smaller than the adjacent area, a statistically significant cold spot will be formed, which is called the cold spot area, that is, the area with low ESV change value.

#### 2.3.4. The Gravity Model

The gravity model is derived from physical concepts [69]. Later, it was widely used in trade [70], energy [71], transportation [72] and other fields. This research adopts the center of gravity shift model, which can intuitively reveal the spatial evolution characteristics of the center of gravity of ESV.

If the study area consists of n units (n represents administrative units), and ($x_{i}$,$y_{i}$) is the geometric coordinate of the *i*th unit (*I* = 1, 2, 3,..., *n*), then the barycenter coordinate of ESV is ($\bar{x},\bar{y}$), it can be expressed as [73]:

(10) $$\bar{x}=\frac{\sum_{i=1}^{n}m_{i}x_{i}}{\sum_{i=1}^{n}m_{i}}$$

(11) $$\bar{y}=\frac{\sum_{i=1}^{n}m_{i}y_{i}}{\sum_{i=1}^{n}m_{i}}$$

The movement direction of the center of gravity can be expressed as:

(12) $$\theta =\left[\frac{k\times \pi }{2}+\tan^{-1}\left(\frac{\bar{y_{t_{2}}}-\bar{y_{t_{1}}}}{\bar{x_{t_{2}}}-\bar{x_{t_{1}}}}\right)\right]\times \frac{180{}^{\circ}}{\pi }$$

The movement distance of the center of gravity is calculated by the following formula:

(13) $$D=\sqrt{{\left(\bar{x_{t_{2}}}-\bar{x_{t_{1}}}\right)}^{2}+{\left(\bar{y_{t_{2}}}-\bar{y_{t_{1}}}\right)}^{2}}$$

where θ represents the deflection angle of the center of gravity of ESV, ($x_{t_{1},}y_{t_{1}}$) and ($x_{t_{2}},\;y_{t_{2}}$) respectively represent the coordinates of the center of gravity at the beginning and end. $t_{1},\;\;t_{2}$ represents the start time and end time, and k is the adjustment coefficient. *D* represents the moving distance of the center of gravity.

## 3. Results

### 3.1. Land Use Changes during the Period 1995–2015

**Figure 2:**
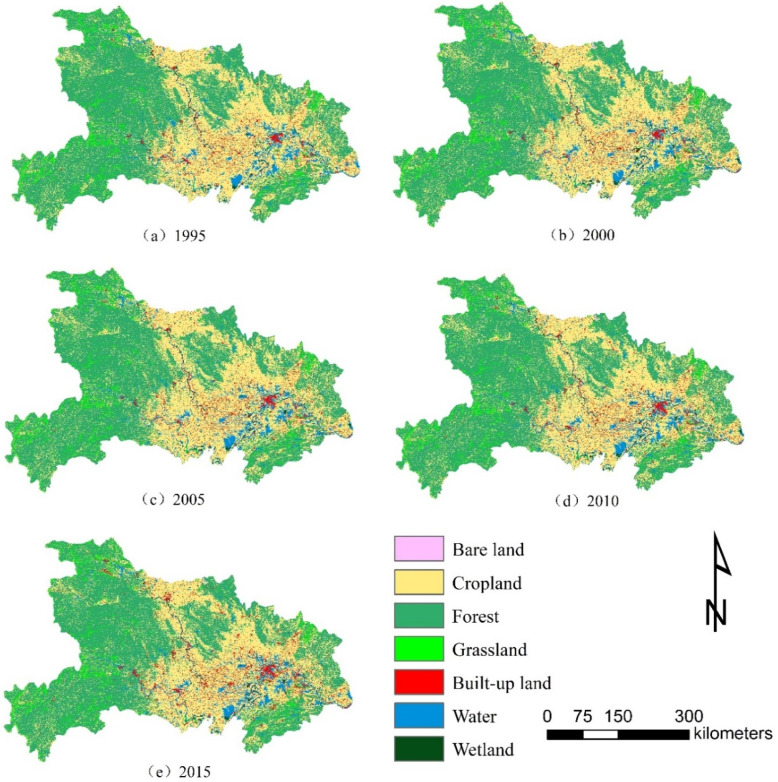
Land use change from 1995 to 2015.

After reclassifying land use sensing data of Hubei province in five periods from 1995 to 2015, the land use type distribution map is obtained (Figure 2). According to the results, from 1995 to 2015, forest land and cropland were the main land use types in Hubei province, and the built-up land mainly concentrated in Wuhan city and the center of each urban area in central Hubei province was in an inverted “herringbone” shape in the trend of water flow along the river at the center of Wuhan. There was an expanding trend toward wetland mainly along both sides of the Yangtze River and Han River. The water area was mainly distributed in the relatively flat Jianghan Plain.

The area and change of each land use type in the study area in five periods were obtained by zoning statistics of the reclassified data (Table 2). On the whole, the significant expansion of the built-up land and the reduction of cropland and forest were the most significant characteristics of land use change in Hubei province in the past 20 years. During the past 20 years, the area of the built-up land increased the most, which was 2148.49 km^2^, and the area increased sharply from 2010 to 2015, accounting for more than 70% of the total increased area. The area of cropland decreased the most, reaching 2047.36 km^2^, and the area decreased accounted for nearly 60% of the total area decreased from 2010 to 2015. Hubei province covered the largest area of forest, followed by cropland.

According to the land use transfer matrix (Table 3) from 1995 to 2015, the characteristics of land use transformation in Hubei province were as follows: (1) From 1995 to 2015, the area of cropland and forest land eventually decreased. Cropland was mainly converted into forest land and the built-up land, with an area of 12,598.13 and 4323.12 km^2^, respectively. Forest land was mainly converted into cropland and grassland, with an area of 12,996.31 and 3356.11 km^2^, respectively. (2) Although water areas and wetlands had been developed and used for 20 years, they eventually increased. In terms of land use types occupied, waters occupied the largest area of cropland at 3450.73 km^2^, and wetland also occupied the largest area of cropland at 526.92 km^2^. The increase in the area of water areas and wetlands may be related to the policy of “returning farmland to lakes” implemented in the middle reaches of the Yangtze River. (3) The area of the built-up land had increased the most in 20 years, mainly occupying 4323.12 km^2^ of cropland and 862.23 km^2^ of forest land. The occupied land area was mainly used for urban construction. The built-up land was the land use type with the most thorough artificial transformation and the most affected by urbanization. The difference between the area transferred to the built-up land and the area transferred from the built-up land was the largest, which are 5729.23 and 3576.65 km^2^, respectively. Therefore, the expansion of the built-up land was the most dramatic during the study period. The occupation of crop land and forest land while the expansion of the built-up land became a significant land use transformation feature in the period of rapid urbanization in Hubei Province. During the study period, with the rapid development of urbanization and the increase of urban and rural incomes, the demand for housing, travel and consumption has increased, and the proportion of secondary and tertiary industries in Hubei province has increased, which has promoted the occupation of urban and rural built-up land to cropland, forest land and other ecological land.

### 3.2. Temporal Response of Ecosystem Service Values to Land Use Change

#### 3.2.1. Changes in Ecosystem Service Values of Different Land Use Types

Driven by the rapid urbanization development in Hubei province, land use transition has led to the fluctuation of ecosystem services value (Table 4). As a large amount of cropland and forest land were occupied by the built-up land expansion, the ecosystem services value of cropland and forest land showed a decreasing trend during the study period, in which the loss of forest land was the largest (USD 1.00 billion), and the loss of cropland was the largest (2.96%). From 1995 to 2015, the ESV of water area and wetland increased by USD 2.03 billion and 1.20 billion, respectively, with the largest increment of water area, but the largest increment of wetland, reaching 56.90%. Overall, the total value of ESV in Hubei province increased by USD 1.84 billion in 20 years, and the total ESV showed an increasing trend from 1995 to 2010. From 2010 to 2015, when the expansion of the built-up land invaded cropland and forest land was the most serious, the loss of ESV in Hubei province was nearly USD 320 million. The policy of returning cropland to lake in the middle reaches of the Yangtze River in 1998 may be responsible for the increase of ecosystem services value of water area and wetland, while the rapid development of urbanization may be responsible for the loss of ecosystem services value of cropland and forest land.

#### 3.2.2. Changes in Individual Ecosystem Service Values

According to land use and land cover change and ESV coefficient, the contribution of each ecosystem function caused by land use transition to the overall ESV was investigated (Table 5). From the services value of each single ecosystem, hydrological regulation, climate regulation, soil conservation and biodiversity maintenance contribute the most to the overall ESV in the study area. Since 1995, with the rapid development of urbanization in Hubei province, the expansion of the built-up land has occupied a large number of cropland and forest land, and the problem of cropland conversion has intensified, which inevitably leads to the weakening of the ecosystem service function of cropland and forest land. From 2010 to 2015, food production, raw material production, climate regulation, gas regulation, soil conservation and nutrient supply, as the main ecosystem service functions of cropland and forest land, suffered losses to varying degrees, totaling USD 0.50 billion, accounting for the overall ESV losses of the main position. This indicated that the sharp decrease of cropland and forest land caused by rapid urbanization was the main source of ESV loss in the study area. From the perspective of the four service functions of the ecosystem, the largest loss was the regulation service, with a total loss of USD 270 million, followed by the supporting service, with a loss of USD 120 million, and the supply service had a relatively small loss, with a total loss of USD 110 million. In conclusion, the urbanization driven land use transition in Hubei province had the greatest impact on ecosystem regulation services, and the single ecosystem service function of the greatest impact was climate regulation. As the main ecosystem service function of waters and lakes, water supply and hydrological regulation have been increasing, with an increase of USD 250 million and 1790 million, respectively, in the past two decades. The temporal change of ecosystem services value is directly affected by land use transition. The drastic change of land use structure leads to the tightening of resources and environment in the study area, which breaks the balance of original ecosystem and leads to the decline in corresponding ecosystem service function of the region.

### 3.3. Spatial Response of Ecosystem Service Values to Land Use Transformation 

**Figure 3:**
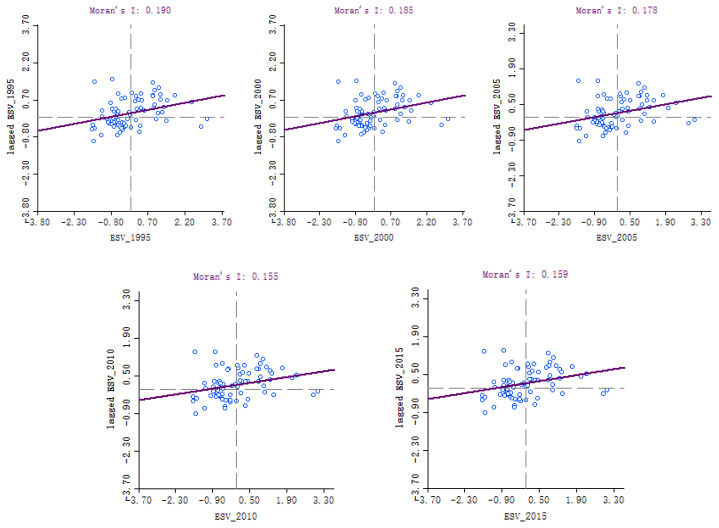
Global Moran’s I in Hubei province from 1995 to 2015.

To explore the spatial response from ecosystem services value to land use transition, Global Moran’s I statistic was used to measure spatial autocorrelation based on factor location and attribute to value. The spatial correlation of ecosystem services value in Hubei province in the past 20 years was analyzed. As shown in Figure 3, Moran’s I values from 1995 to 2015 were greater than 0, and the county units in Hubei province were mostly in the first and third quadrants, indicating that there was a positive spatial correlation between ecosystem services value of Hubei province from 1995 to 2015. Spatial distribution had a certain spatial correlation and presented spatial aggregation distribution pattern. 

**Figure 4:**
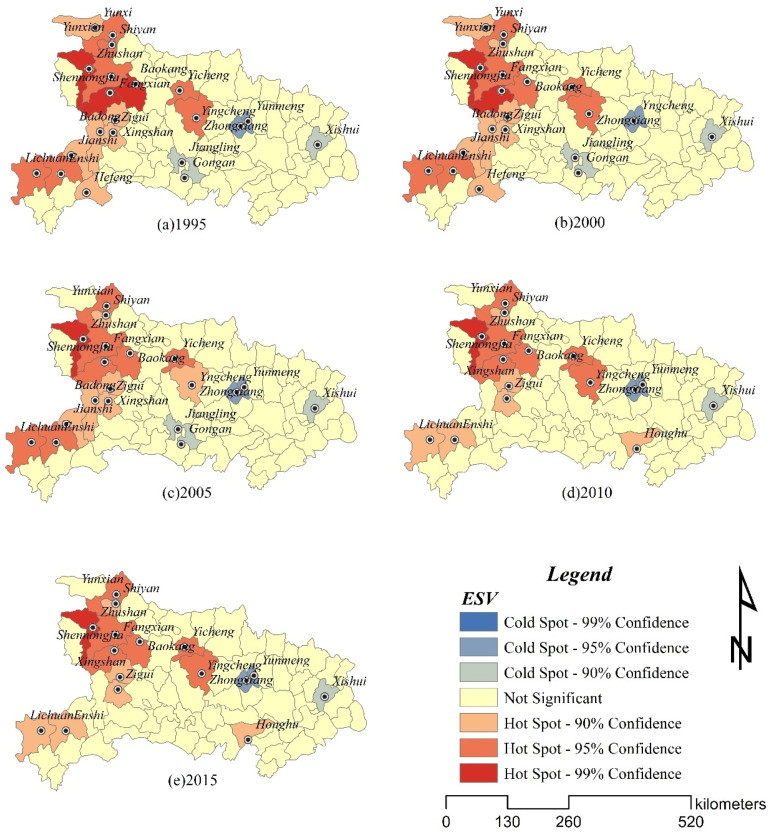
Hot spot analysis in Hubei province from 1995 to 2015.

The hot spot analysis (Formula (7)**–**(9)) was used to reveal the local expression of the change of ecosystem service value in space. The hot spot analysis results (Figure 4) showed that the hot spots of ecosystem service value from 1995 to 2015 are mainly located in the western region where urbanization is relatively slow and rich in forest resources, and the distribution area is large and contiguous. Shennongjia Forestry District in the west was always a hot spot area with 99% confidence. The forest land in Shennongjia Forestry District accounts for more than 85%, the population is small, and the impact of human activities on the ecosystem is small. Fang County, Yun County, Zhushan County, Xingshan County in the northwest, and Lichuan County and Enshi City in the southwest were hot spots, with confidence levels greater than 90%, and they made a great contribution to the overall ESV. Most of these areas are mountainous areas with relatively high forest coverage and good ecosystem conditions. Xishui County in the east and Zhongxiang City and Yunmeng County in the middle were cold spots of ecosystem services value. During this period, the pace of urban construction in this area has accelerated, industry has developed rapidly, and construction land has expanded significantly.

From the number of cold spots and hot spots, from 1995 to 2005, the hot spots with 99% confidence and 95% confidence in Hubei Province decreased, and the cold spots did not change. From 2005 to 2015, the 95% confidence hot spots and 90% confidence hot spots in Hubei Province increased, and Jiangling and Gong’an counties with 90% confidence in the central region were no longer cold spots. Jiangling County and Gong’an County are close to the Yangtze River. During this period, the government paid attention to the prevention and control of water pollution and the protection of the ecological environment, so the ecosystem condition has improved. From the results of the hot spot analysis, in areas with slow urbanization development, the expansion of construction land is not obvious, and the original vegetation coverage is relatively high, so the ecosystem status is relatively stable. In addition, the Yangtze River passes through the territory of Hubei Province. Strengthening the protection of the Yangtze River will help the ecosystem in the Yangtze River Basin to improve. 

**Figure 5:**
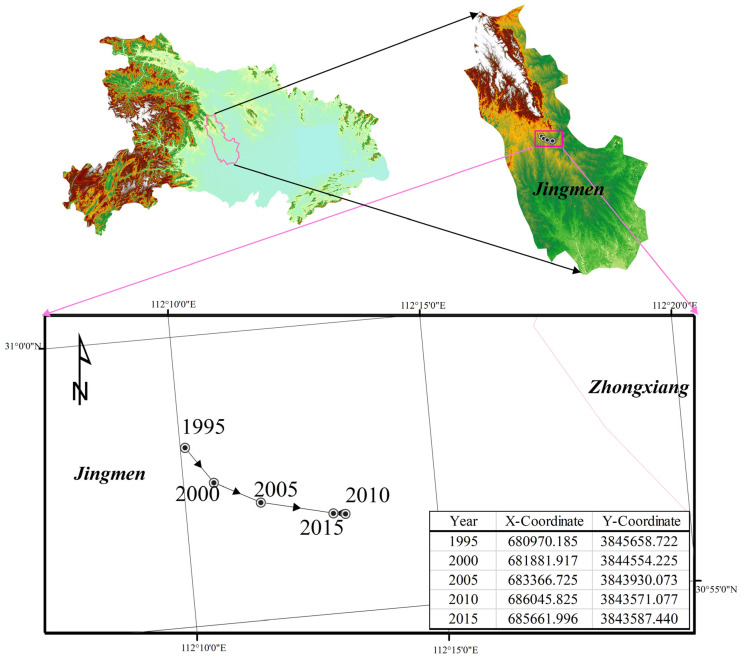
The trajectory of the center of gravity of ecosystem services value in Hubei province from 1995 to 2015.

The calculation of geographical gravity center can help to more accurately grasp the temporal and spatial pattern of ESV from a dynamic perspective. As shown in Figure 5, From 1995 to 2010, the gravity center of ESV shifted from northwest to southeast, with an offset angle of 23.5° and an offset distance of 5.099 km. From 2010 to 2015, the rapid development of urbanization in Hubei province led to a sharp decrease in cropland and forest land, which reduced the value of ecosystem services. The center of ecosystem services value shifted in the opposite direction, with an offset angle of 2.5°, but the deviation distance was not large. During the study period, the focus of ESV was within the boundary of Jingmen City. The results showed that the provision of ecosystem services in Hubei province showed a positive trend from 1995 to 2010, and the ecology in the eastern region was recovering. The ecosystem situation in Hubei province rebounded from 2010 to 2015, which was consistent with the change of ecosystem service values in Hubei province.

## 4. Discussion

Under the background of rapid urbanization development in Hubei province, this study explored the characteristics of land use transformation by using land use transfer matrix and evaluated ESV of the study area. On this basis, the ways of ESV responding to land use transformation in time and space were analyzed. There has been much research on ESV responses to land use overturning changes, but little is known about ESV responses driven by rapid urbanization [33,60,74]. By constructing the analytical framework of “urbanization—land use transformation—ecosystem services value response”, the study further simulates this “social economic—nature” process.

Hubei province, as an important part of China’s key urban agglomeration—the middle reaches of the Yangtze River urban agglomeration, is a typical representative of China’s rapid urbanization development. Under the background of rapid urbanization, a large number of labor force migrate to urban areas, regional economy develops rapidly, urban and rural income increases substantially, and demand for housing and consumption surges [75], thus promoting the occupation of urban and rural built-up land to cropland and forest land. The encroachment of the built-up land on cropland and forest land leads to the increase of the intensity and frequency of human activities’ interference to the ecosystem, which directly threatens the service functions of the ecosystem [76,77]. These functions are mainly food production, climate regulation, soil conservation and so on.

As an ecological security barrier in the Shennongjia Forestry District in Hubei Province, lakes and waters in the Yangtze and Han River basins have made outstanding contributions to the overall ESV. Therefore, in future development planning, the background advantages of the ecosystem should be fully maintained and the negative impact of urbanization on land use transformation and the ecosystem should be weakened. The Yangtze River Protection Law just passed on 26 December 2020, is the first river basin law in China to protect the ecosystem of the whole Yangtze River Basin, which reflects the attention paid by the state and society to the ecological environment of the Yangtze River Basin. The tourism industry in Shennongjia Forestry District is relatively developed. While developing the tourism industry, we must always place “protecting ecological balance” first. Judging from the difference between the eastern, central and western parts of Hubei Province, the western part of Hubei Province is mostly mountainous areas, with relatively low levels of urbanization, high forest coverage, and high ecosystem security capabilities. Therefore, the ESV of each county is also high. From 1995 to 2015, the central region of Hubei Province accelerated urbanization, with rapid expansion of construction land, mostly in plain areas, with low forest coverage, so the average ESV in the central region was the lowest. The eastern region has the highest level of urbanization development, but the river system here is well−developed and contributes a lot to the overall ESV, so the average ESV in the eastern region is at a medium level. From the perspective of ESV hot spots, the distribution is relatively concentrated and the area is large in the past 20 years. Therefore, the synergistic effect of hot spots can be continued and enhanced, and ecological policies such as “encouraging social capital to participate in ecological protection and restoration policies” [78] and “ecological protection compensation policy” [79] can be used to further support the enhancement of local ecological effects. The main goal of cold spot development is to coordinate the contradiction between economic development and ecological protection. The level of urbanization in Hubei Province is still in the upgrading stage, and the built-up land is still in the expansion stage. It is a major challenge for Hubei province to ensure the economic development while maintaining the ecosystem service function and value. Hubei Province can rely on abundant higher education resources and combine its own scientific research advantages to provide intellectual support for enhancing the service functions of the ecosystem. When making plans, we should weigh various factors to avoid improper utilization of resources. We can incorporate the local index of “Ecosystem services value” into the local government performance assessment. For the cold spot area, we should first identify the reasons for the low ecosystem services value. Then, according to the degree of cropland occupied by the expansion of the built-up land, the degree of river pollution and whether the land layout is reasonable, formulate targeted plans, such as strictly controlling the building density, appropriately increasing the proportion of green space, increasing forest reserves, etc. Enhancing people’s understanding of the relationship between with ecological environment and the ecological management of government departments [80] are of great significance for realizing regional sustainable development [81] and steadily improving the capability of ecosystem service assurance.

## 5. Conclusions

In this study, taking Hubei province in central China as the research area, we analyzed the characteristics of land use transformation driven by urbanization and the response of ecosystem service value to land use transformation from 1995 to 2015 at the county scale. The results showed that:

(1) Urbanization is an important inducement of land use transformation in Hubei province. From 1995 to 2015, with the continuous improvement of urbanization level in Hubei province, the land use structure changed constantly, and the continuous expansion of the built-up land occupying a large amount of cropland and forest land became the most significant feature of land use transformation. In addition, affected by the policy of “returning farmland to lakes” in the Yangtze River Basin, the area of water area and wetland increased during the study period, second only to construction land.

(2) The spatial and temporal distribution pattern and evolution of ecosystem services value are directly affected by land use transformation. During the study period, the value of ecosystem services in Hubei province increased from USD 118.08 billion in 1995 to USD 119.93 billion in 2015, showing an overall trend of growth. However, from 2010 to 2015, a large number of cropland and forest land in the study area were transformed into the built-up land, resulting in a total ESV loss of USD 480 million for food production, raw material production, climate regulation, gas regulation, soil conservation and other single ecological functions, which became the main reason for the ESV loss in the study area. Therefore, paying close attention to the development trend of urbanization and relevant policies, timely optimizing the land use structure and controlling the area of ecological land occupied by construction land is the focus of the work of relevant government departments in the future. The well−developed water system in Hubei province and the rich forest resources in the west have contributed greatly to the overall ESV of Hubei province. Strengthening the protection of the forest areas in the west and the waters of the Yangtze River and Han River is of great significance to maintaining the stability of the ecosystem service value in Hubei province.

(3) Affected by rapid urbanization and land use changes, the ecosystem services value in Hubei province was high in the west and low in the middle and the east. The hot spots were mainly concentrated in the western regions with slow urbanization development and high vegetation coverage, while the cold spots were mainly distributed in the central regions with strong interference from human activities. In the last five years, when the expansion of the built-up land was the most drastic, the center of gravity of ESV broke the original deviation direction, showing a rebound of migration. Using a variety of spatial analysis methods to analyze the development trend and spatial characteristics of ecosystem services value can help us formulate differentiation policies according to the actual situation of different regions, so as to help the local improve the ecological environment and enhance the ecosystem services value. In general, the response of ecosystem services value to land use transformation is the result of the interaction and synergistic development of the complex system of “society−economy−nature” driven by urbanization.

## Figures and Tables

**Table 1:** Ecosystem services value coefficients per unit area in Hubei (USD·hm^−2^·a^−1^).

Top Level Ecosystem Services	Second Level of ES	Cropland	Forest	Grassland	Wetland	Bare Land	Water
Provisioning service	Food production	394.96	98.74	90.84	201.43	1.97	244.87
	Raw material produce	118.49	229.08	135.60	197.48	5.92	82.94
	Water supply	−31.60	118.49	75.04	1022.94	3.95	2812.10
Regulating service	Gas Regulation	185.63	753.38	476.58	750.42	25.67	339.66
	Climate regulation	142.18	2254.22	1259.92	1421.85	19.75	663.53
	Purifying Environment	43.45	660.57	416.02	1421.85	80.97	1129.58
	Hydrological Regulation	434.45	1475.17	922.89	9569.84	47.39	21,600.27
Supporting service	Soil conservation	233.03	917.29	580.59	912.35	29.62	185.63
	Maintaining nutrient supply	51.34	70.11	44.76	71.09	1.97	13.82
	Biodiversity	47.39	835.34	527.93	3108.32	27.65	1295.46
Cultural service	Aesthetic Landscape	23.70	366.32	233.03	1868.15	11.85	548.99
	A total of	1643.03	7778.70	4763.20	20,545.73	256.72	28,916.87

**Table 2:** Changes in the area of each category from 1995 to 2015.

Land Use Type	Periods	Cropland	Forest	Grassland	Water	Wetland	Bare Land	Built-Up
Area (km^2^)	1995	69,066.83	93,458.77	7542.89	9797.99	1027.49	51.77	4954.25
	2000	69,425.60	92,660.73	7541.91	9619.07	1663.92	47.80	4940.98
	2005	68,603.72	92,591.53	7506.02	10,437.53	1513.55	46.80	5200.84
	2010	68,234.93	92,534.27	7486.07	10,435.48	1613.12	47.80	5548.33
	2015	67,019.47	92,167.33	7450.26	10,499.27	1612.13	48.79	7102.74
Area ratio (%)	1995	37.15	50.27	4.06	5.27	0.55	0.03	2.67
	2000	37.35	49.84	4.06	5.17	0.90	0.03	2.66
	2005	36.90	49.81	4.04	5.61	0.81	0.03	2.80
	2010	36.71	49.78	4.03	5.61	0.87	0.03	2.98
	2015	36.05	49.58	4.01	5.65	0.87	0.03	3.82
Variation (km^2^)	1995−2000	358.76	−798.04	−0.98	−180.63	636.43	−3.98	−13.28
	2000−2005	−821.87	−69.20	−35.89	820.32	−150.37	−1.00	259.87
	2005−2010	−368.80	−57.25	−19.96	−2.00	99.57	1.00	347.49
	2010−2015	−1215.45	−366.94	−35.81	63.87	−0.99	1.00	1554.41
	1995−2015	−2047.36	−1291.43	−92.63	701.56	584.64	−2.98	2148.49
Rate of change (%)	1995−2000	0.52	−0.85	−0.01	−1.84	61.94	−7.68	−0.27
	2000−2005	−1.18	−0.07	−0.48	8.51	−9.04	−2.08	5.26
	2005−2010	−0.54	−0.06	−0.27	−0.02	6.58	2.13	6.68
	2010−2015	−1.78	−0.40	−0.48	0.61	−0.06	2.08	28.02
	1995−2015	−2.96	−1.38	−1.23	7.14	56.90	−5.76	43.37

**Table 3:** Transition area matrix of LULC from 1995 to 2015 (km^2^).

Land Use Type (km^2^)		2015						
		Cropland	Forest	Grassland	Water	Wetland	Bare Land	Built-Up
1995	Cropland	47,475.51	12,598.13	743.47	3540.73	526.92	11.98	4323.12
	Forest	12,996.31	75,296.37	3356.11	1021.90	114.76	7.98	862.23
	Grassland	834.29	3307.21	3233.36	96.80	12.97	4.99	67.86
	Water	2687.48	802.35	99.80	5275.17	525.92	0.00	423.13
	Wetland	252.48	40.92	5.99	285.41	399.18	0.00	44.91
	Bare land	13.97	4.99	2.99	1.00	0.00	20.96	7.98
	Built−up	2903.04	311.36	22.95	300.38	35.93	2.99	1398.15

**Table 4:** ESVs for different land use types in Hubei Province from 1995 to 2015 (USD).

Ecosystem Type	Period	Cropland	Forest	Grassland	Water	Wetland	Bare Land	Total
ESV (Billion)	1995	11.35	72.70	3.59	28.33	2.11	0.00	118.08
	2000	11.41	72.08	3.59	27.82	3.42	0.00	118.31
	2005	11.27	72.02	3.58	30.18	3.11	0.00	120.16
	2010	11.21	71.98	3.57	30.18	3.31	0.00	120.25
	2015	11.01	71.69	3.55	30.36	3.31	0.00	119.93
ESV Variety (Billion)	1995−2000	0.06	−0.62	0.00	−0.52	1.31	0.00	0.23
	2000−2005	−0.14	−0.05	−0.02	2.37	−0.31	0.00	1.85
	2005−2010	−0.06	−0.04	−0.01	−0.01	0.20	0.00	0.08
	2010−2015	−0.20	−0.29	−0.02	0.18	0.00	0.00	−0.32
	1995−2015	−0.34	−1.00	−0.04	2.03	1.20	0.00	1.84

**Table 5:** ESVs for different functions in Hubei Province from 1995 to 2015 (USD).

Top Level Ecosystem Services	Second Level of ES	ESV (Billion)					ESV Variety				
		1995	2000	2005	2010	2015	95–00	00–05	05–10	10–15	95–15
Provisioning service	FP	3.98	3.99	3.98	3.96	3.91	0.01	−0.02	−0.01	−0.05	−0.07
	RMP	3.16	3.16	3.15	3.15	3.13	0.00	−0.01	0.00	−0.02	−0.04
	WS	3.81	3.81	4.03	4.04	4.05	0.00	0.22	0.01	0.02	0.25
Regulating service	GR	9.09	9.08	9.08	9.07	9.02	−0.01	−0.01	0.00	−0.05	−0.07
	CR	23.80	23.70	23.70	23.69	23.59	−0.10	0.00	−0.01	−0.10	−0.20
	PE	8.04	8.06	8.12	8.13	8.10	0.02	0.06	0.01	−0.02	0.06
	HR	39.63	39.75	41.33	41.39	41.42	0.12	1.57	0.06	0.03	1.79
Supporting service	SC	10.90	10.89	10.86	10.85	10.79	−0.01	−0.03	−0.01	−0.06	−0.11
	MNS	1.06	1.06	1.06	1.06	1.05	0.00	0.00	0.00	−0.01	−0.01
	MB	10.12	10.23	10.28	10.30	10.27	0.11	0.05	0.02	−0.03	0.15
Cultural service	AL	4.49	4.57	4.59	4.60	4.59	0.08	0.01	0.02	−0.01	0.09
	Total	118.08	118.31	120.16	120.25	119.93	0.23	1.85	0.08	−0.32	1.84

Note: FP, food production; RMP, raw material production; WS, water supply; GR, gas regulation; CR, climate regulation; PE, purifying environment; HR, hydrological regulation; SC, soil conservation; MNS, maintaining nutrient supply; MB, maintaining biodiversity; AL, aesthetic landscape.

## Data Availability

All data generated or analyzed during this study are included in this published article.
